# Quality Decision-Making Practices in Pharmaceutical Companies and Regulatory Authorities: Current and Proposed Approaches to Its Documentation

**DOI:** 10.1007/s43441-020-00167-7

**Published:** 2020-05-29

**Authors:** Magdalena Bujar, Neil McAuslane, Patricia Connelly, Stuart R. Walker

**Affiliations:** Centre for Innovation in Regulatory Science (CIRS), 160 Blackfriars Road, London, SE18EZ UK

**Keywords:** Regulatory, Decision making, Decision-making documentation, Knowledge management

## Abstract

**Background:**

Pharmaceutical companies and regulatory agencies endeavor to relate their decision making with outcomes to improve future decision making and to ensure that gained knowledge is fed back into a learning system. Nevertheless, such a correlation can only be achieved by documenting the expected outcome of a decision at the time it is made, enabling comparison of the expected outcome with the actual result.

**Methods:**

Participants at an international workshop discussed how the documentation of decisions could be evolved as companies and agencies look to improve their knowledge base. Discussions were informed by a pre-workshop survey of pharmaceutical companies and regulatory agencies.

**Results:**

Most survey participants from 12 companies (55% response rate) and 11 agencies (73% response) have a system in place to enable documentation of major decisions, however, systems are used primarily to document outcomes rather than the process, while information from documentation is not always used, and feedback loops are not in place. The majority of participants indicated that their organization currently documents most decision-making practices included in the proposed template. Workshop participants agreed that all major past decisions should be referenceable and suggested incentives to enable decisions to be referenced, and confirmed elements and characteristics of a decision-documentation template.

**Conclusions:**

This survey and workshop identified the current landscape and gaps in the documentation of decision making and suggested revisions for a proposed documentation template. The use of technology to enable information extraction with support from artificial intelligence and future decision making was a recommendation highlighted by participants.

## Introduction

**Figure 1.**
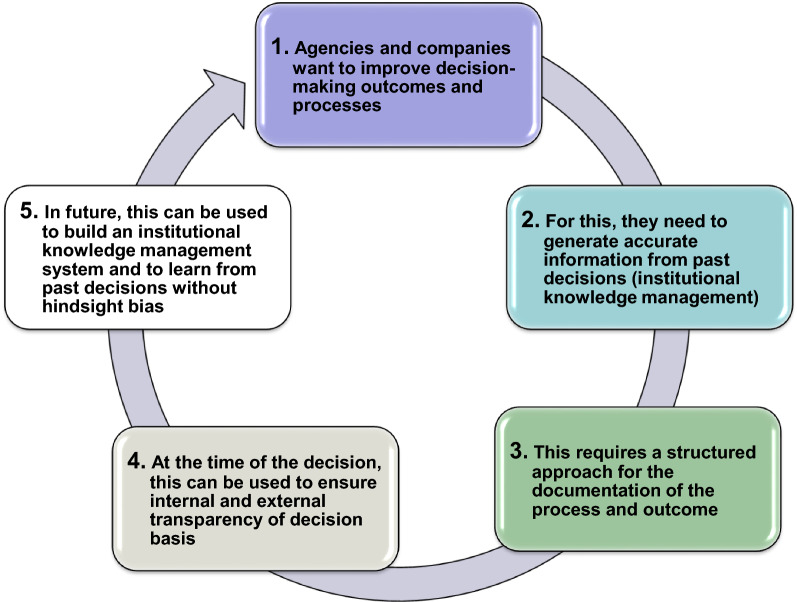
Improving future decision making through decision-making documentation.

Pharmaceutical companies and regulatory agencies continually endeavor to improve their internal decision-making practices in order to ensure that quality is built into the process and to guarantee that accurate information from past decisions is available to inform current and future decisions (Fig. 1). As part of this continuous improvement process, these organizations seek to relate their decision making to the outcomes, a correlation that can only be achieved by documenting the expected outcome of a decision at the time when it is made. This timely documentation enables a comparison of the expected outcome of a decision with the actual result and determines the impact without “hindsight bias”; that is, the tendency to rationalize past decision making based on current knowledge [1]. Thus, documentation can help improve future decision making and is a way of ensuring that gained knowledge is fed back into a learning system, or what may be called “institutional knowledge management.”

The importance of institutional knowledge management, supported by the documentation of decision making is being recognized within regulatory agencies and pharmaceutical companies as well as in other types of agencies such as the National Aeronautics and Space Administration (NASA) [2] as well as other industries. The US Food and Drug Administration (FDA) recently initiated the Knowledge-aided Assessment & Structured Application (KASA) system to gather and control information about new drugs during their lifecycle [3] and Dr Janet Woodcock, Director of the Center for Drug Evaluation and Research, a center of the U.S. Food and Drug Administration, emphasized that “Accurate historic information from many past drug reviews is essential to informing current and future reviews—and to assure consistent regulatory decision-making” [4].

In its decade-long research program into quality decision making, the Centre for Innovation in Regulatory Science (CIRS) has evaluated and developed decision-making frameworks and tools. [5–10] As part of this program, participants at a 2017 CIRS workshop identified areas of importance in decision making as (1) establishing the context or frame of the decision; (2) performing an assessment of linked or similar decisions; (3) identifying possible and expected outcomes now and in the future; and (4) ascertaining areas of uncertainty. To ensure that these processes occur; however, the considerations used to make decisions must be transparent and a record trail or documentation must be developed that details not just the decision outcome, but also the decision basis, both of which are critical to inform future decisions [11]. In 2018, CIRS initially explored the topic of decision-making documentation by companies at a Technical Forum, where a survey was undertaken. Survey results highlighted challenges faced by companies in documenting decision making, including the documentation of decisions rather than decision processes, and the lack of visibility between therapeutic area teams within companies.

CIRS wished to further explore how systematic structured approaches to decision making and the documentation of decisions could be utilized or evolved as companies and agencies look to improve their institutional knowledge base to facilitate timely, linked, consistent, and informed decisions. Accordingly, the organization conducted a survey of pharmaceutical companies and regulatory agencies and organized a workshop which reviewed approaches to better decision making in companies and agencies through documentation, quality decision-making practices and knowledge management. This paper brings together the outcomes of the survey and the workshop with the aim of identifying current approaches and a template to document decision making.

## Methods

### Objectives

#### Survey Objectives

In order to provide a framework for discussions at the workshop, CIRS conducted a focused survey of pharmaceutical companies and regulatory agencies to

- identify companies’ and agencies’ current approaches to the documentation of decision making for major decisions during the development and review of medicinal products;
- investigate the perceived value of the documented information regarding the decision and how it is used by companies and agencies;
- determine what organizations believe should be documented at the time of the decision to correlate the decision at the time with the actual outcome and to utilize the learnings for institutional knowledge management to improve future decision making.

#### Workshop Objectives

Group discussions at the workshop covered several topics with specific objectives.

Group 1 was asked to

- consider which decisions should be referenceable when making current or future decisions within companies and agencies and why; and
- suggest possible incentives or systems that would encourage or enable decisions to be referenced and ensure that the information is usable and retrievable, so as to maximize company and agency knowledge management processes.

Group 2 was tasked with

- discussing potential elements and characteristics of a template that could be used to document information of value to inform future and current decisions.

Group 3’s objective was to

Discuss public assessment reports and their potential as knowledge management tools for other stakeholders in understanding an agency’s decision making.

#### IRB Approval

This programme of research was exempt from the national research ethics committee approval, as it poses minimal risk, as defined by IRB, and is educational in nature. In addition, the article does not contain any clinical studies with human or animal subjects performed by any of the authors.

#### Survey Methods

The pilot and main survey were purposive sampling studies; invitations were issued via email.

##### Pilot Study

The survey developed to inform the workshop was pilot tested with organisations that had been invited to the meeting, namely major pharmaceutical companies and regulatory agencies. The purpose was to verify the format, clarity, and usability of the survey. A total of three agencies and three companies were approached, and two of each agreed to participate in the pilot in March 2019. Participants were asked

1. In general, how did you find the questionnaire?
2. Is the format of the questionnaire clear? (yes/no; If no, please specify)
3. Is the language used in the questionnaire clear? (yes/no; If no, please specify)
4. Are there any questions you found difficult to answer? (yes/no; If yes, please specify)

Overall, pilot results were positive, and participant comments were used to make minor questionnaire revisions.

##### Main Study

The main study ran from April–May 2019. The questionnaire was sent to 22 major pharmaceutical companies that were CIRS members and to 15 regulatory agencies that were either considered major international regulatory agencies or that had been invited to the workshop. The questionnaire covered major decisions within each organization and had three sections on (1) current practice, (2) a proposed approach for documentation, and (3) future areas for consideration. Agency and company questionnaires were analogous where appropriate.

#### Workshop Methods

At the June 2019 workshop, focus groups led by a group chair discussed topics relative to decision-making documentation. The results of each group’s discussion were reported to the entire workshop by a rapporteur.

Group 1 consisted of 5 representatives from pharmaceutical companies; 2 from regulatory agencies; 1 from a health technology assessment agency; 1 from a decision-making consultancy group; and 3 from CIRS.

Group 2 consisted of 4 representatives from pharmaceutical companies; 4 from regulatory agencies; 1 from a health technology assessment agency; 1 from an academic institution; 1 from a non-profit consortium to improve the drug development process; and 3 from CIRS.

Group 3 comprised 6 representatives from pharmaceutical companies; 4 from regulatory agencies; 1 from an academic institution; 1 from a non-profit organization; and 2 from CIRS.

## Results

### Survey Results

Responses were received from 12 (55% response rate) pharmaceutical companies, all in the top 20 according to research and development budgets, and 11 (73% response rate) regulatory agencies in the US (Food and Drug Administration; FDA), EU (European Medicines Agency, EMA), UK (Medicines and Healthcare products Regulatory Agency, MHRA), Switzerland (Swissmedic), Netherlands (Medicines Evaluation Board, MEB), Sweden (Medical Products Agency, MPA), Canada (Health Canada), Australia (Therapeutic Goods Administration, TGA), Israel (Ministry of Health, MoH), South Africa (South African Health Products Regulatory Agency, SAHPRA) and Turkey (Türkiye İlaç ve Tıbbi Cihaz Kurumu, TITCK). The focus of responses was on major decisions such as the decision to submit a medicine for regulatory review or the decision to approve a medicine.

### Current Practice

Most of the responding agencies (10/11) and all 12 companies have a system in place to enable documentation of decision making of major decisions. These systems are mostly formal as opposed to informal (8 agencies, 8 companies), as defined by standard operating procedures and guidelines. The organizations document the decisions made (9 agencies, 11 companies); the evidence considered (9 agencies, 8 companies), but it is important to note that only some document the actual decision-making process (6 agencies, 4 companies).

The most important steps that enabled the implementation of existing documentation systems within each organisation were management and organizational buy-in. Other enablers included the organizational desire to make the decision-making process more robust and consistent and improve data quality, effectiveness, and access. The existence of an external champion (an individual from outside the organization who will initiate and support the implementation of a documentation system) was seen as an important driver. Among companies, templates and project management systems were the approaches most frequently cited as enabling the systems and for agencies, most important approaches were templates and document management systems.

All agencies and companies indicated that the documentation of decision making is mandatory and is supported through education, training, and peer review of reports detailing the decision making. Importantly there are no incentives for most of the organizations to ensure that documented information is utilized. However, in general, participants reported that it is an expectation that they use the information during day-to-day operations, but no feedback mechanism exists to ensure this use, except indirectly through individual performance processes.

**Figure 2.**
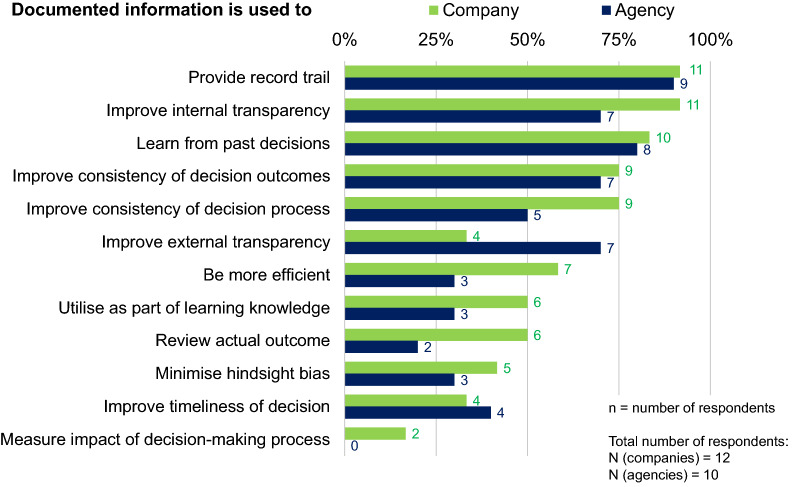
Pharmaceutical company and regulatory agency purposes for documenting decision making.

Incentives for the documentation for an organization should be driven by the desire to have a more consistent and robust decision-making process and to improve external transparency. Indeed, the four main reasons for the use of these systems identified by companies and agencies were the provision of a record trail, the improvement of internal transparency, learning from past decisions, and the improvement of the consistency of decision outcomes (Fig. 2).

The majority of agency participants (7/9) agreed or strongly agreed that their organization’s current documentation system is fit for purpose, while in companies, an equal number of participants agreed (5/12) whereas some were not sure (5/12) of the appropriateness of their organization’s system. Although agency responders largely (6/8) believed that companies were transparent in their documentation of all the relevant information on which they have based their decision making regarding the development of a medicine, the majority of companies (6/10) did not agree that agencies were transparent regarding all the relevant information on which they based their decision in the review of a medicine. In general, participants were unsure as to whether or not their organisations’ decision-making documentation systems will evolve in the future.

Agency comments regarding the future evolution of documentation systems included indications that it was expected that the system would become formalized or made more efficient; by for example improving searchability, sharing information in a structured manner, automating the process using technology or introducing customer relationship management. Company participants commented that in future the system would include improved tools, templates, and processes.

### A Proposed Approach for Documentation

Based on previous research [5–10], CIRS provided participants with a list of decision-making practices that should be documented and participants were asked to indicate which of these elements were part of record-keeping practice within their organization.

**Figure 3.**
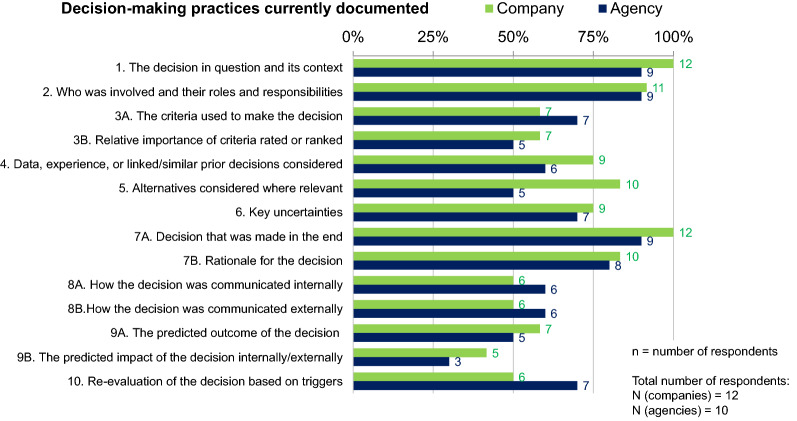
Percentage of elements from a proposed decision-making documentation template that are currently documented by pharmaceutical companies and regulatory agencies.

The elements of decision making that were least frequently documented by agencies and companies were the predicted internal/external impact of the decision (documented by 30% of the agencies and 42% of the companies) and the predicted outcome of the decision and the relative importance of the criteria rated or ranked (both documented by 50% of the agencies and 58% of the companies). Respondents agreed that it would be appropriate for their organization to document those elements that currently are not documented (Fig. 3).

All companies and most agencies agreed that the proposed approach for documentation could be helpful for documenting decision making in organizations. Survey participants specified other areas that could be included in the proposed approach to documentation, including an assessment of key risks and their relevance as well as an assessment of the probability of success. Uncertainties in the external environment were also named as a potential element of the approach. As one respondent explained

“There are factors outside of the control of the decision makers that could affect both the expected outcome and impact of the decision. These factors, if known, should be documented and considered as part of future evaluations of the decision. If not, then the decision could be questioned in the future and falsely suspected for failure.”

Survey participants provided important challenges and their solutions to decision-making documentation (Table 1).

**Table 1.** Challenges and Solutions to Decision-Making Documentation According to Pharmaceutical Companies and Regulatory Agencies.

Challenges	Solutions
A lack of effective templates, user-friendly information technology systems and software to facilitate data input/extraction and searchability, as well as no agreement as to who should perform documentation, which is necessary for consistency, and who should use the information	Update infrastructure and technology as well as processes, practices, and templates to improve the searchability of past decisions, increase flexibility to review and update the information through, for example, Periodic Benefit-Risk Evaluation Report (PBRER) and minimize potential sources of bias/assumptions
A large volume of information and number of decisions as well as the changing nature of those decisions and their applicability	Develop novel approaches to decision making, documenting decisions, and knowledge management to convert the volume of decisions into an asset for improved effectiveness and efficiency of decision making, making them universal to support harmonization
The high administrative burden involved with templates, checklists, and maintaining a document management system and staff turnover, contrasted by the need for an acceptance by top management to effectively implement systems by increasing resources	Create culture and incentives to promote team-based integration and establish processes and procedures for interdisciplinary teams with clear roles and responsibilities as well as quality assurance/feedback loops to ensure transparency and consistency
A lack of awareness of the principles and importance of documenting not just decisions but also the decision process as well as a lack of case studies to demonstrate how documentation systems are used in practice by companies/agencies and the efficiencies gained or lost through such systems	Increase awareness though training and education regarding the principles of decision making and documentation and socialization to support change management

## Workshop Discussion Results

### Discussion Group 1

#### Referenceable Decisions and Their Value to Current and Future Decisions

This discussion group agreed that given the scientific complexity, risk profile, and other critical factors and variables present throughout the product lifecycle, it may be difficult to determine the magnitude of all factors in decision making and which of these might be referenceable with a high degree of certainty. It was therefore agreed that all substantive information from past decisions regarding quality, safety, and efficacy should be referenceable. Even minor decisions that appear to be low risk and inconsequential could act as a catalyst for major issues and deficiencies.

#### Uncertainty

Uncertainty was a key topic for this discussion group. They indicated that each decision has uncertainties and that these need to be documented, particularly to be able to re-evaluate the decision with new information. Referencing former US Secretary of Defense Donald Rumsfeld’s well-remembered quote about “known and unknown unknowns” [12], the discussion group cited examples of “known unknowns” or uncertainties that are beyond the regulatory assessment of quality, safety, and efficacy in factors such as population demographics, lifestyle, healthcare systems, clinical practice, and environmental factors. The value and impact of “unknown knowns” such as a manufacturing or sterilization process are well understood but not consciously considered. “Unknown unknowns,” in which decision makers are not aware of what they do not know, constitute factors that are unexpected, based on existing knowledge or experience.

#### Barriers

Barriers to the documentation of decision making that were discussed were similar to those uncovered through the survey and included the lack of a retrievable/searchable system that provides the ability to readily search, and functionally utilize content contained within documents; the lack of structured and standardized content; and the need to balance a return on investment against technical feasibility and resource allocation. An organizational culture that encourages maintenance of the status quo or that regards assessments as individual intellectual achievements can represent another obstacle to documentation as does the need to mitigate legal liability while presenting decisions and their corresponding rationale in a clear and concise manner.

#### Incentives

The ability to look back and apply knowledge management from past decisions and extrapolate to future decision making is a significant incentive to document decision making, leveraging past efforts to eliminate redundancies and streamline processes. It is advisable; however, to broaden the strategic application of past decision making from a product to a policy level. In this way, past decision making can be referenced in guidelines and may be equally applicable and beneficial to all stakeholders. Policy makers are advised to provide practical examples of the acceptable application of this past knowledge.

#### Recommendations

The group made several recommendations to facilitate decision-making documentation:

- Make novel, precedent-setting issues easily accessible, searchable, indexed, and given increased weight and oversight. This is critical, as these decisions are complex and the sample size that is used to make these decisions is typically too limited to develop overall guidance.
- Document the rationale for decisions; consider all factors available at the time of the decision in relation to new experience and information obtained post-decision, and discover if original decision makers would make different determinations with new information.
- Develop case studies of divergent regulatory decisions including the rationale, interpretations, and other considerations for decisions.
- Categorize negative decisions as outcomes and opportunities for “lessons learned” rather than as mistakes; regarding them as opportunities to inform decision making given new information/evidence and methodologies.
- Develop structured, standardized content; leverage technology and data standards/informatics, artificial intelligence, analytics, taxonomy, and ontological frameworks.
- Research cultural incentives to document decision making; that is, internal incentives such as professional development and external cultural incentives such as communication of uncertainty and promotion of awareness to healthcare stakeholders, including patients, citizens, and healthcare professionals. This communication can serve to ensure trust and transparency and mitigate potential criticism as well as to provide an opportunity for proactive patient engagement.

### Discussion Group 2

This group reviewed and discussed the proposed decision-making documentation template in the context of various agency and company decision-making scenarios to integrate a high degree of quality into decisions, based on items listed in Fig. 3 that were previously surveyed.

There was strong consensus within the group that the proposed template is valuable in a variety of settings as it facilitates a common understanding across all stakeholders, presents a clear and objective picture of why a decision was made, and relieves users of the need to search for important information through volumes of data. In the case of litigation, it provides a record of what was done and why the use of the template can result in efficiencies in future similar decision making, the facilitation of the implementation of new policies at a health authority, and the ability for authorities into use reliance approaches where one agency would leverage the decision of another agency in the approval of medicines.

However, the group agreed that the most appropriate decision framework may vary depending on the situation, with some commonalities as well as some significant differences. They therefore recommended the addition of some nuances or elaboration to the proposed model, for example by adding notes for each item to be documented.

This discussion group recommended the changing of the template item that called for the ranking of the relative importance of decision-making criteria as it was considered unlikely that an explicit, quantitative ranking would be feasible and recommended that the template allow for documentation of the importance of criteria and, such as unmet need, and how these are considered and factored into the decision. It was suggested that items 8–10 within the template may be more related to decision implementation and used in exceptional circumstances. They further recommended splitting item 6 into known and unknown uncertainties and the addition of “key influencers” to decision-making rationale documentation that would be used to capture important decision drivers that may otherwise not be recorded such as the industry need to win market share or satisfy investment milestones or requirements.

Steps in the Development of a Decision-Making Documentation Template:

1. Initial development of a template items based on previous research [5–10]
2. Survey assessment to determine the current practice for documentation of template items (Fig. 2)
3. Modifications to draft template based on workshop discussions:
  Splitting the template into two parts (process and implementation of decision)
  Addition of notes for each item
  Modification of items 3 (removal of rating/ranking of criteria), 6 (splitting into known and unknown uncertainties) and 7 (inclusion of “additional” influences from draft template

**Figure 4.**
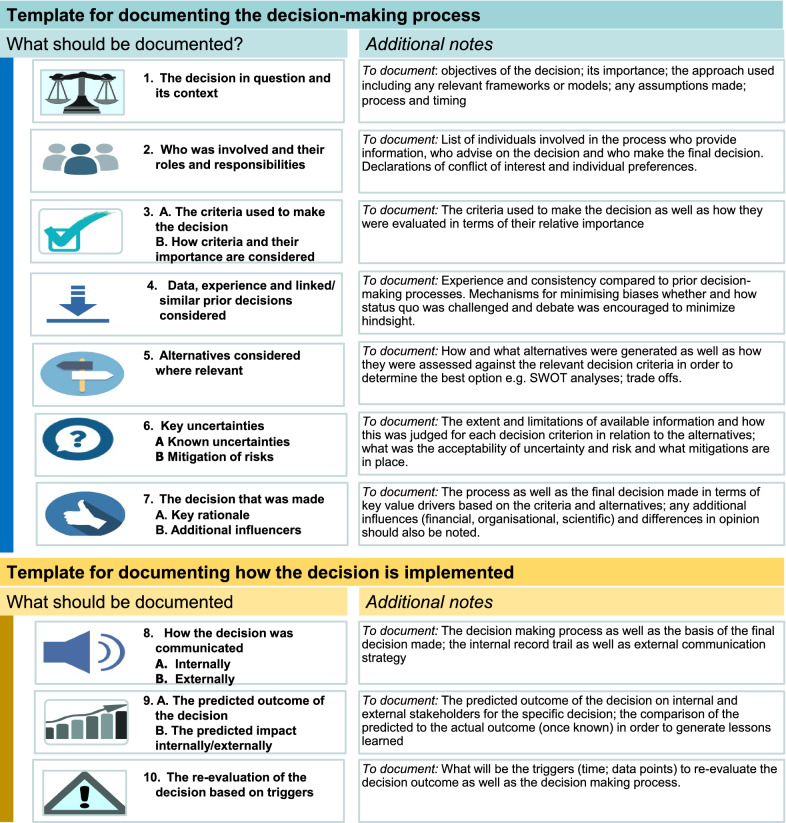
The revised documentation template for decision making.

The final revised template is shown in Fig. 4.

### Discussion Group 3

This group discussed the role of public assessment reports as knowledge management tools for stakeholders such as other regulatory authorities, health technology assessment agencies, companies, and patients in understanding an agency’s decision making and to suggest potential improvements. The group agreed that regulatory agencies do not clearly articulate how decisions are made and that uncertainties and decision comfort levels are not always defined. In addition, details of the benefit-harm balance of the evaluated medicine should be explicit and principles of quality decision making should be considered; however, there may be legislative or cultural barriers that impede the enhancement of public assessment reports.

Discussion participants recommended the endorsement of a common template for a harmonized executive public assessment report summary appropriate for agencies, payers, and patients. It was further suggested that this summary use standardized headings to improve transparency, aid in understanding of the decision rationale and help in agency comparison and assist emerging economies placing reliance on reference agencies. Headings could include proposed therapeutic indication, conditions of use, treatment modalities evaluated, medical need, criteria used to make the decision and their relative importance, experience and linked/similar prior decisions considered, current therapeutic resources, alternatives considered, key uncertainties, ultimate decision and key rationale, risk management plan and patient feedback. The group proposed that a survey of agencies that produce public assessment reports be conducted in which it could be determined if these proposed headings could be used and to identify other possible headings. Finally, agencies were encouraged to consider publishing public assessment reports or releasing information related to negative decisions or the extension of indications.

**Figure 5.**
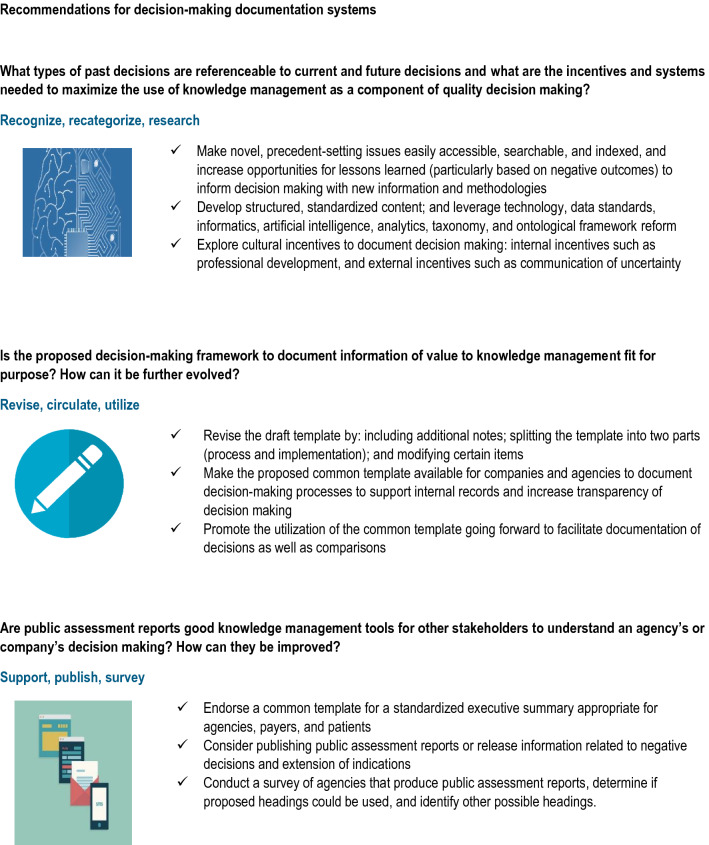
Recommendations for the evolution of the documentation of decision making from three workshop discussion groups.

The combined recommendations of the three workshop discussion groups for documentation systems are listed in Fig. 5.

## Discussion

This research explored how systematic structured approaches to decision making and the documentation of decisions could be enhanced and used by companies and agencies to improve their institutional knowledge base to facilitate timely, linked, consistent, and informed decisions.

With the increased role of systematic, structured frameworks and their use to document what organizations consider in making decisions such as benefit-risk assessments [13, 14], large amounts of aggregated knowledge have been accumulated within and across stakeholders as to how specific decisions have been reached. In previous research in quality decision making, the value of maintaining an audit trail for important decisions was considered critical for decision-making, as transparency in the process and the potential for better predictability in future judgments were linked to having a record/audit trail of previous successes [5]. This record keeping, along with better electronic systems to capture the information, is becoming an important part of many companies’ and agencies’ knowledge management library. However, the critical information that should be distilled from the documentation of the decision process and the subsequent outcomes to help inform and improve future decisions remain to be determined. Furthermore, while many decisions are made within companies and agencies every day, choosing the type of decisions (major or minor) that need to be referenceable to inform other decisions could be debated. This workshop and pre-workshop survey sought to determine which decisions are key to document and what should be recorded by whom as well as the barriers and incentives to document decisions and to find and utilize the information for current and future determinations.

The majority of survey respondents and workshop participants indicated that decision-making documentation takes place within their organization, mostly through formal, mandatory systems. The mandatory nature of the systems is reflective of the “buy-in” for documentation among organizational leadership, which was indicated as a primary enabler of decision-making documentation by both survey and workshop participants. However, there are no incentives for most of the organizations to ensure that documented information is utilized. In general, participants reported that it is an expectation that they use the information during day-to-day operations, but no feedback mechanism exists to ensure this use, except indirectly through individual performance processes.

The majority of these organizational systems already contain most of the elements in the CIRS-proposed documentation template. Notable exceptions to this for both groups were in the documentation of internal and external communication of the decision and the predicted outcome and impact of the decision. In addition, most workshop participants felt that although the criteria and their importance should be included there was agreement to revise this item from the template to exclude ranking or rating of the criteria as this may not be feasible.

Most agency survey participants felt that the documentation system in their organizations was fit for purpose, whereas companies were more divided as to the functionality of their organization’s current systems. Agencies largely indicated that their documentation systems were transparent, but the majority of companies did not agree that agencies were transparent in this regard. In fact, criticisms of public assessment reports, the vehicle through which most agencies communicate the bases of their regulatory decisions, have included their lack of transparency and difficulty in access [15, 16]. However, public assessment reports have recently undergone improvements at many regulatory agencies and all agencies participating in the survey reported that they expected documentation of decision making to continue to evolve in their organization. The workshop discussion group tasked with discussing public assessment reports made several suggestions to facilitate this evolution, including the creation of executive summaries and the use of standardized headings to aid in transparency and understanding.

Workshop discussants agreed that all decisions were potentially referenceable and as such need to be documented. They also agreed that the proposed decision-making documentation template was of value, but made some revisions, including that of “additional influencers” so that factors such as competitive intelligence could be documented as being part of a company’s decision making.

The efficient use of technology was highlighted by both survey respondents and workshop participants for its importance in the evolution of decision-making documentation; however, it has been specified both by workshop participants and researchers that this efficient use requires standardization and documentation [17–19]. As reported by De Grave, “systematic, structured documentation is instrumental to ensure that right information is at hand to be able to reach/extract information from sources and support self-learning capabilities” [20].

## Future Research

In addition to future directions described as a result of the discussion group recommendations, the authors suggest the following future research for the development of the template.

- It would be of value to explore further lessons learned from other agencies such as NASA and businesses, such as the manufacturing industry, in terms of the value and approaches used for documentation of decision making. This information could be used to further optimize the template.
- As patients are one of the major beneficiaries of the template, particularly with regard to agency decision making, it would be of interest to review this template with patient organizations. This would aim to ensure that the template contains the information that patients perceive as important in understanding the decision making of organisations involved in the development, review, and reimbursement of medicines.
- Once the template is finalized, the next step would be to pilot it in companies and agencies to evaluate the practicality and applicability of the template, as well as its value in improving the quality and transparency of decision-making processes within agencies and companies.

## Conclusions

The documentation of key strategic decision-making processes is important for ensuring a written record and for internal and external knowledge sharing. Since 2011, CIRS has undertaken research and developed tools in this area and some of this work is still in progress but more will be required.

The outcomes of the survey and workshop identified the current landscape and gaps in the documentation of decision making. Utilization of the documented information was cited as a particular challenge by participants. This research also confirmed the value of and suggested revisions to the proposed template for documentation. In addition, the study outcome was a recommendation for the use of this revised template as an element of best practice in decision making.

Finally, one of the important solutions highlighted by both the survey and workshop discussions was the use of technology to facilitate the easier extraction of information and to underpin future decisions. In addition, the use of technology could also support self-learning or artificial intelligence capabilities to facilitate the implementation of systems for improved decision making and its documentation.
